# Multiple gene editing in porcine embryos using a combination of microinjection, electroporation, and transfection methods

**DOI:** 10.14202/vetworld.2022.2210-2216

**Published:** 2022-09-16

**Authors:** Quynh Anh Le, Manita Wittayarat, Zhao Namula, Qingyi Lin, Koki Takebayashi, Maki Hirata, Fuminori Tanihara, Lanh Thi Kim Do, Takeshige Otoi

**Affiliations:** 1 Bio-Innovation Research Center, Tokushima University, 7793233 Tokushima, Japan; 2 Laboratory of Animal Reproduction, Faculty of Bioscience and Bioindustry, Tokushima University, 7793233 Tokushima, Japan; 3 Faculty of Veterinary Science, Prince of Songkla University, 90110 Songkhla, Thailand; 4 Department of Veterinary Medicine, College of Coastal Agricultural Sciences, Guangdong Ocean University, 524088 Guangdong, China; 5 Department of Animal Theriogenology and Surgery, Faculty of Veterinary Medicine, Vietnam National University of Agriculture, 100000 Hanoi, Vietnam

**Keywords:** clustered regularly interspaced short palindromic repeats/Cas9, electroporation, microinjection, porcine zygotes, transfection

## Abstract

**Background and Aim:**

Mosaicism – the presence of both wild-type and mutant alleles – is a serious problem for zygotic gene modification through gene editing using the Clustered regularly interspaced short palindromic repeats-Cas9 (CRISPR/Cas9) system. Different delivery methods, such as microinjection (MI), electroporation (EP), and transfection (TF), can be used to transfer CRISPR/Cas9 components into porcine zygotes. This study aimed to develop a method that combines MI, EP, and TF to improve mutation efficiency mediated through the CRISPR/Cas9 system for a triple-gene knockout in pigs.

**Materials and Methods:**

The study consisted of three groups: The MI group with three simultaneously microinjected guide RNAs (gRNAs) targeting α-1,3-galactosyltransferase (*GGTA1*), cytidine 32 monophosphate-N-acetylneuraminic acid hydroxylase (*CMAH*), and β-1,4-N-acetyl-galactosaminyltransferase 2 (*B4GALNT2*); the MI + EP group with two gRNAs targeting *GGTA1* and *B4GALNT2* genes delivered into zygotes through MI, followed by EP of gRNA targeting the *CMAH* 1 h later; and the MI + EP + TF group with MI of gRNA targeting *GGTA1* gene into zygotes, followed by EP of gRNA targeting *CMAH* 1 h later, and then TF of gRNA targeting the *B4GALNT2* gene into zona-free zygotes after another hour.

**Results:**

The rate of blastocysts carrying mutations in one or two gene(s) was significantly higher in the MI + EP + TF group than in the MI group. However, the blastocyst formation rate of zygotes in the MI + EP + TF group was lower than that of the zygotes in the other treatment groups.

**Conclusion:**

The combination of CRISPR/Cas9 delivery methods may improve the mutation efficiency of triple-gene edited porcine blastocysts.

## Introduction

To date, genetically modified models of pig-to-human organ and cell transplantation (xenotransplantation) have become the focus of many researchers to overcome the shortage of donor organs [1]. However, one of the main obstacles that limit the performance of xenotransplantation is the hyperacute rejection of xenografts between two discordant species [2]. Hyperacute rejection is related to genes such as α-1,3-galactosyltransferase (*GGTA1*), cytidine 32 monophosphate-N-acetylneuraminic acid hydroxylase (*CMAH*), and β-1,4-N-acetyl-galactosaminyltransferase 2 (*B4GALNT2*) [3–6]. To solve this problem, *GGTA1/CMAH/B4GALNT2* triple-gene knockout pigs should be produced for pig-to-human xenotransplantation.

Clustered regularly interspaced short palindromic repeats-Cas9 (CRISPR/Cas9) is a gene-editing technology often used to introduce targeted modifications in pigs [7, 8]. Different delivery methods, such as microinjection (MI), electroporation (EP), and transfection (TF), can be used to transfer CRISPR/Cas9 components into porcine zygotes. However, each method has different procedures, benefits, and disadvantages [9–11]. The MI combined with EP has been used to introduce DNA into several types of organisms, including embryonic mouse kidney explants, to enhance the transgene expression compared with that for each method alone [12, 13]. In porcine zygotes, CRISPR/Cas9-mediated multiple gene editing using MI followed by EP increases biallelic mutation rates [14]. A combination of different delivery methods has the potential to improve gene-editing efficiency in zygotes or embryos. However, our previous study targeting *GGTA1* demonstrated no increase in mutation efficiency after double MI of CRISPR/Cas9 system into porcine zygotes before and after *in vitro* fertilization [11]. Mechanical damage caused by the two rounds of micromanipulation procedures may cause reduced viability of successfully injected zygotes, resulting in no increase in mutation efficiency. A combination of EP and LF instead of repeated rounds of MI procedure may reduce mechanical damage to the zygotes. Thus, to increase the rates of biallelic mutation and reduce the effect of each delivery method in a triple-gene knockout in porcine blastocysts, a combination of MI, EP, and TF methods could be a viable approach.

Therefore, this study aimed to improve the methods for delivering CRISPR/Cas9 reagents into zygotes to generate *GGTA1/CMAH/B4GALNT2* triple-gene knockout porcine blastocysts and examined whether the combination of MI, EP, and TF methods could improve the rates of total mutation and total biallelic mutation in the resulting blastocysts. The data will provide new insights for advancement in the current methodology for the production of multiple gene knockout animals.

## Materials and Methods

### Ethical approval

The animal experiments were approved by the Institutional Animal Care and Use Committee of Tokushima University (approval number: T28-21).

### Study period and location

This study was conducted from October 2020 to May 2021. All laboratory works were conducted at the Bio-Innovation Research Center of Tokushima University and Laboratory of Animal Reproduction, Faculty of Bioscience and Bioindustry, Tokushima University.

### Oocyte collection, *in vitro* maturation (IVM), and *in vitro* fertilization (IVF)

Oocyte collection, IVM, and IVF were performed as described previously [15]. Pig ovaries were collected from the prepubertal gilts at a local slaughterhouse. Cumulus-oocyte complexes were collected and cultured in a maturation medium for 44 h. The matured oocytes were coincubated with frozen-thawed ejaculated spermatozoa (1 × 10^6^ cells/mL) for 5 h in porcine fertilization medium (Research Institute for the Functional Peptides Co., Yamagata, Japan) and subsequently cultured in porcine zygote medium (PZM-5; Research Institute for the Functional Peptides Co.) until cytoplasmic MI, EP, and Cas9-guide RNA (gRNA) ribonucleoprotein complex (RNPs) TF. Oocytes were incubated in a humidified incubator at 39°C with 5% CO_2_.

### Design of gRNA sequence

Alt-R CRISPR crRNAs and the tracrRNA system supplied by Integrated DNA Technologies (IDT; Coralville, IA, USA) were used for gRNA. In the present study, we selected three target genes: *GGTA1, CMAH*, and *B4GALNT2* (Table-1). *GGTA1, CMAH*, and *B4GALNT2* encoding enzymes mediate the generation of xenogeneic antigens and need to be inactivated to prolong organ survival after xenotransplantation [6, 16]. The CRISPR direct web tool (https://crispr.dbcls.jp/) was used to design the gRNA sequences [17]. To minimize off-target effects, the COSMID web tool (https://crispr.bme.gatech.edu/) [18] was used to confirm that the 14 nucleotides at the 3' end of the designed gRNAs matched only the target regions of each gene.

**Table-1:** gRNA and primer sequences used for sequencing analysis.

Target gene (Chromosome localization*)	gRNA				Primer	
	
	Target sequence	PAM	Target	Strand	Forward primer	Reverse primer
*GGTA1* (Chromosome 1, NC_010443.4.)	AGACGCTATAGGCAACGAAA	AGG	Exon 2	Sense	AAAAGGGGAGCACTGAACCT	ATCCGGACCCTGTTTTAAGG
*CMAH* (Chromosome 7, NC_010449.4.)	GAAGCTGCCAATCTCAAGGA	AGG	Exon 1	Sense	GCTGTCAATGCTCAGGGATT	TGCCAAACCTAATTGGGAGA
*B4GALNT2* (Chromosome 12, NC_010454.3.)	TTGAGGATCGACAGACATCT	AGG	Exon 2	Antisense	GACCAGACATCGTTCCCAGT	GGGAACTGGCTGTAAAGTGG

* Based on NCBI: Sus scrofa isolate TJ Tabasco breed Duroc, whole genome shotgun sequence, Sscrofa11.1 (GCF_000003025.5)

### Cytoplasmic MI

Cytoplasmic MI was performed as previously described [11]. Briefly, CRISPR/Cas9 components were injected into zygotes in a 20 mL drop of PZM-5 covered with mineral oil. Nuclease-free duplex buffer (Integrated DNA Technologies) containing 100 ng/μL of gRNA(s) and 100 ng/μL of Cas9 protein (Takara Bio, Inc., Shiga, Japan) was loaded into the injection pipette (Femtotips II, Eppendorf, Hamburg, Germany) and injected into the cytoplasm through air pressure using a microinjector (FemtoJet 4i; Eppendorf). After MI, the embryos were cultured in PZM-5 in a humidified incubator containing 5% CO_2_, 5% O_2_, and 90% N_2_. On day 3 after fertilization (day 0), all of the cleaved embryos were subsequently cultured in a porcine blastocyst medium (PBM; Research Institute for the Functional Peptides Co.) for 4 days.

### Electroporation

Electroporation was performed as previously described [7]. Briefly, zygotes were electroporated (five 1 ms pulses at 25 V) with a nuclease-free duplex buffer containing 100 ng/μL of gRNA and 100 ng/μL of Cas9 protein. After EP, embryos were cultured in PZM-5 and PBM as described above.

### Cas9-gRNA RNPs TF

Lipofection-mediated RNP TF was performed as previously described [9]. Briefly, zygotes were freed completely from their zona pellucida (ZP) after exposure to 0.5% (w/v) actinase-E (Nissui Pharmaceutical, Tokyo, Japan) for 20–30 s. The RNP TF solution was prepared by diluting 2 μL of jetCRISPR (Polyplus-TF, Illkirch-Graffenstaden, France) with nuclease-free duplex buffer (IDT) containing 167 ng/μL of gRNA and 500 ng/μL of Cas9 protein to a final volume of 30 mL. After 15 min of incubation, the RNP TF solution was added to 470 μL of PZM-5 containing ZP-free embryos and then coincubated for 5 h. After 5 h of incubation, the embryos were cultured in PZM-5 and PBM, as described above.

### Analysis of the targeted gene in embryos

Genomic DNA was isolated from blastocysts collected individually, and the genomic regions flanking the gRNA target site were polymerase chain reaction (PCR) amplified using specific primers (Table-1). The PCR products were extracted using agarose gel electrophoresis, and the genotype of each blastocyst was analyzed using Sanger sequencing, followed by the application of the tracking of indels by decomposition bioinformatics package [19] as described previously [10]. Blastocysts were classified as having biallelic mutations (carrying no wild-type [WT] sequences), mosaics (carrying more than 1 type of mutation and the WT sequence), or WT (carrying only the WT sequence). Total mutation rate was defined as the ratio of the number of gene-edited blastocysts to the total number of sequenced blastocysts. The biallelic mutation rate in each target gene was defined as the proportion of blastocysts with biallelic mutations to the total number of sequenced blastocysts.

### Experimental design

We introduced gRNAs targeting *GGTA1, CMAH*, and *B4GALNT2* genes with Cas9 protein into *in vitro*-fertilized zygotes through MI or in combination with EP and RNP TF to evaluate the effects of these methods, when used alone or in combination for delivering CRISPR/Cas9 components, on the blastocyst formation rate and efficiency of target mutations in the resulting blastocysts.

Our previous studies have confirmed the efficiency of each gRNAs targeting *GGTA1*, *CMAH*, and *B4GALNT*2 introduced into zygotes using EP [20–22]. The efficiency of gRNAs targeting *GGTA1* and *B4GALNT2* was also evaluated using the MI technique [11, 22]. These gRNAs were used primarily for MI. Gene-editing efficiency using RNP TF is usually low [9, 23]; therefore, gRNA targeting *B4GALNT2*, which has confirmed high gene-editing efficiency using MI and EP [22], was used for RNP TF. The processing time for each method was determined as previously reported [9, 22, 24]. The order of the gene-editing treatment was determined according to our previous studies. Performing MI before EP is better at reducing mortality in porcine zygotic gene editing than vice versa [14]. The MI and EP are generally performed in the pronuclear phase [25]; however, RNP lipofection-mediated gene editing requires a 5 h exposure time. Therefore, the order of gene-editing treatments was MI, EP, and RNP TF.

Zygotes were randomly assigned to three treatment groups. One of which three gRNAs and Cas9 protein was introduced into the zygotes using MI 12 h after the start of IVF (MI group). In the second group (MI + EP group), two gRNAs targeting *GGTA1* and *B4GALNT2* genes were introduced into the zygotes using MI 11 h after the start of IVF, and gRNA targeting the *CMAH* gene was introduced into the zygotes using EP 1 h later. In the third group (MI + EP + TF group), gRNA targeting *GGTA1* gene was introduced into the zygotes through MI 11 h after the start of IVF, gRNA targeting the *CMAH* gene was introduced into the zygotes through EP 1 h later, and gRNA targeting the *B4GALNT2* gene was introduced into ZP-free zygotes through TF 1 h later. The gRNAs were added at a concentration of 100 ng/μL.

### Statistical analysis

All percentage data were subjected to arcsine transformation and then analyzed using analysis of variance, followed by Fisher’s protected least significant difference test. The percentages of edited and biallelic blastocysts in the total number of blastocysts were analyzed using Chi-square analysis with Yates’ correction. StatView software (Abacus Concepts, Berkeley, CA, USA) was used for the statistical analysis. Differences with p = 0.05 or less were considered statistically significant.

## Results

**Figure-1:**
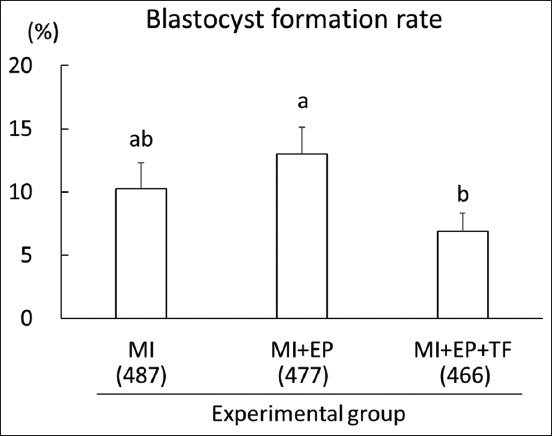
Effects of combination of microinjection, electroporation, and transfection on blastocyst formation rate of porcine zygotes. MI: Three gRNAs targeting *GGTA1, CMAH*, and *B4GALNT2* genes were simultaneously introduced into zygotes using MI 12 h after the start of *in vitro* fertilization (IVF). MI + EP: Two gRNAs targeting *GGTA1* and *B4GALNT2* genes were simultaneously introduced into zygotes using MI 11 h after the start of IVF, and then, gRNA targeting *CMAH* gene was introduced into zygotes through EP 1 h later. MI + EP + TF: gRNA targeting *GGTA1* gene was introduced into zygotes using MI 11 h after the start of IVF, gRNA targeting *CMAH* gene was introduced into the zygotes through EP 1 h later, and gRNA targeting *B4GALNT2* gene was then introduced into the zona-free zygotes through TF 1 h later. Nine replicates were analyzed for each treatment group. The numbers within parentheses under the X-axis indicate the total number of examined zygotes. Error bars indicate mean ± SEM. ^a,b^Bars with different letters differ significantly (p < 0.05). MI=Microinjection, EP=Electroporation, TF=Transfection.

As shown in Figure-1, the blastocyst formation rate (6.6%) of zygotes treated with the MI method in combination with EP and TF (MI + EP + TF group) was significantly lower (p < 0.05) than that 12.8% of zygotes treated with a combination of MI and EP methods (MI + EP group). However, the blastocyst formation rate (10.1%) of zygotes treated with the MI method alone (MI group) was similar to that of zygotes treated with the other methods.

**Figure-2:**
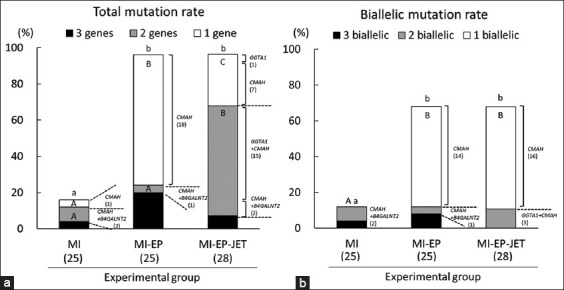
Mutation rates of blastocysts derived from zygotes edited by combining microinjection, electroporation, and transfection. (a) Total mutation rate of blastocysts. The proportions represent the percentage of blastocysts carrying the mutation number of each target gene in the total examined blastocysts. (b) Biallelic mutation rate of blastocysts. The proportions represent the percentage of blastocysts carrying biallelic mutation events in the total examined blastocysts. MI: Three gRNAs targeting *GGTA1, CMAH*, and *B4GALNT2* genes were simultaneously introduced into zygotes using MI 12 h after the start of *in vitro* fertilization (IVF). MI + EP: Two gRNAs targeting *GGTA1* and *B4GALNT2* genes were simultaneously introduced into zygotes using MI 11 h after the start of IVF, and then, gRNA targeting *CMAH* gene was introduced into the zygotes through EP 1 h later. MI + EP + TF: gRNA targeting *GGTA1* gene was introduced into zygotes using MI 11 h after the start of IVF, gRNA targeting *CMAH* gene was introduced into the zygotes through EP 1 h later, and gRNA targeting *B4GALNT2* gene was then introduced into the zona-free zygotes through TF 1 h later. The details of the edited genes are indicated on the side of the bars. The numbers within parentheses under the X-axis indicate the total number of examined blastocysts. The numbers within parentheses under the names of the edited gene indicate the total number of classified blastocysts. ^a-b^The total rates of blastocysts carrying mutation, irrespective of the number of genetic or biallelic mutations, with different letters differ significantly (p < 0.05). ^A-C^The rates of blastocysts carrying same number of mutations (1–3 genes or 1–3 biallelic) with different letters differ significantly (p < 0.05). MI=Microinjection, EP=Electroporation, TF=Transfection.

Mutation rates evaluated by sequencing each target site in the resulting blastocysts (Figure-2a) showed that the total mutation rate in the MI group (16.0%) was significantly lower (p < 0.05) than that in the other groups (96% and 96.4% in the MI + EP and MI + EP + TF groups, respectively). The rate of blastocysts with one mutation was significantly higher (p < 0.05) in the MI + EP group (72.0%) and in the MI + EP + TF group (28.6%) than that in the MI group (4.0%). Moreover, the rate of blastocysts with two mutations was significantly higher (p < 0.05) in the MI + EP + TF group (60.7%) than that in the MI group (8.0%) and MI + EP group (4.0%). The total biallelic mutation rate in the MI group (12.0%) was significantly lower than that in the other groups (68.0% and 67.9% in the MI + EP and MI + EP + TF groups, respectively) (Figure-2b). Moreover, the rates of blastocysts with one biallelic mutation were significantly higher (p < 0.05) in the MI + EP group (56.0%) and MI + EP + TF group (55.9%) than those in the MI group (0%) (Figure-2b). There was no significant difference in the number of blastocysts with the three mutations among the experimental groups.

## Discussion

The MI is a commonly used method for delivering CRISPR/Cas9 components into the embryonic genome. However, it is technically challenging and has a high embryonic lethality [26, 27]. Therefore, we established gene editing through EP of the Cas9 protein method, in which CRISPR/Cas9 components are introduced into zygotes through EP, which can subsequently cause highly efficient gene modification [7]. Liposome-mediated RNP TF is another method that can be used with ZP-free zygotes [9]. However, each method still has disadvantages; therefore, an alternative methodology could be to combine them all.

One of the possible factors affecting the efficiency of producing gene-knockout animals through the MI method is the maximum volume of the injectable solution into zygotes [28]. Although the maximum volume is approximately 1–5% of the cell volume [29], the capacity of porcine zygotic cells to expand after MI seemed to be lower than that of other species, such as mice, rabbits, and rats, which resulted in lower gene disruption efficiency [28]. Therefore, the combination of MI with other methods can increase the efficiency of multiple gene targeting by reducing the volume of solution injected. Our results support our hypothesis, showing that the rates of total mutations and total biallelic mutations were significantly higher when the combination methods (MI + EP group or MI + EP + TF group) were used.

Introducing engineered endonuclease CRISPR/Cas9 system into embryos using EP resulted in a better embryonic survival rate than that with MI because EP does not cause a cellular disturbance if the protocol is well optimized and established [22, 30–32]. Moreover, EP-mediated multiple gene editing using more than 2 gRNAs simultaneously has statistically no harmful effects on the viability of porcine zygotes [21, 33]. These data are in agreement with our findings, demonstrating that the blastocyst formation rate of zygotes treated with the MI and EP methods was similar to that of zygotes treated with the MI method alone. Although EP increased the total mutation rates, the rates in blastocysts with more than 2 mutations were still low. This may be because the volume of the injectable solution for MI used to target the two genes was greater than the required capacity. Moreover, gene disruption yield can be limited by EP when the gene-editing machinery delivered into cells may not reach the nucleus, due to the difficulty in ZP penetration of large-sized Cas9 [30, 34]. In such cases, the third method may be helpful in overcoming this limitation of MI.

Details of the gene-editing results demonstrated bias in gene-editing efficiency – gRNA targeting *CMAH* has high gene-editing efficiency, but those targeting other genes have low efficiency (Figure-2). Our previous studies confirmed the efficiency of gRNAs targeting *GGTA1* and *B4GALNT2* using both MI and EP [11, 20, 22], and that of gRNA targeting *CMAH* using EP [21]. When these gRNAs were introduced alone using EP, the total gene-editing efficiency, including mosaic genotypes, was over 70%, and the percentage of biallelic mutant embryos without mosaic embryos was approximately 40%. When gRNAs targeting *GGTA1* and *B4GALNT2* were introduced simultaneously using MI (MI + EP group), both the *GGTA1* and *B4GALNT2* genes were hardly edited. However, in the MI + EP + LF group, in which each of the three gRNAs was introduced into the embryo through different methods, there was a dramatic increase in gene editing of the *GGTA1* gene (Figure-2a). We assume that this is due to potential crosstalk interference. Our previous study [21] also demonstrated that multiple gRNAs can result in crosstalk due to each being affected by the presence of other gRNAs, resulting in poor efficiency. It can be inferred that a similar phenomenon occurred in the MI group, in which three gRNAs were introduced simultaneously, resulting in the low multiple gene-editing rates in the MI and MI + EP groups. Therefore, the MI + EP + LF group, in which three gRNAs are introduced using different methods, achieved stable multiple gene editing.

The TF using lipofection has the potential to deliver the gene-editing CRISPR/Cas9 system into ZP-free embryos [9]. Hence, we applied this method as a third approach to improve the system’s efficacy. The results showed that TF combined with MI and EP appeared to increase the rate of blastocysts with the two mutations compared to the other groups. In the present study, TF treatment was performed 11 h after the start of IVF, during which actin filaments became concentrated in both male and female pronuclei [35, 36]. This confirms our previous findings that the success of lipofection-based TF depends on the high number of actin filaments present in embryos [9]. However, the biallelic mutation rates obtained from the MI + EP + TF group were still the same as those obtained from the MI + EP group, suggesting that this system did not work well to generate biallelic mutations. This might be because TF timing was probably earlier before the pronuclear syngamy stage. Gene editing in porcine zygotes using RNP lipofection is still under development and is less efficient than EP or MI [9, 23]. This may be the reason why the mutation rate of *B4GALNT2* edited using the lipofection technique did not improve in the MI + EP + LF group in the present study. We need to optimize the TF timing in future studies.

## Conclusion

The results obtained in the present study can be applied with further steps to improve multiple gene disruption using the CRISPR/Cas9 system. A combination of MI, EP, and TF to deliver CRISPR/Cas9 components contributes to better outcomes in blastocysts with one and two biallelic mutations than MI alone. However, the rates of the three biallelic mutations did not increase significantly. Therefore, some limitations remain and further studies are recommended.

## Authors’ Contributions

QAL, FT, and TO: Conceived the study and wrote the manuscript. QAL: Performed all experiments, collected data, and wrote the original draft of the manuscript. TO: Designed the study, coordinated all experiments, and reviewed the manuscript. MH: Performed sequencing analysis. ZN, QL, KT, and LTKD: Contributed to laboratory work and statistical analyses. MW: Revised the manuscript. All authors have read and approved the final manuscript.

## Data Availability

The datasets used and/or analyzed during the present study are available from the corresponding author on reasonable request.
